# Salt stress under the scalpel – dissecting the genetics of salt tolerance

**DOI:** 10.1111/tpj.14189

**Published:** 2019-01-12

**Authors:** Mitchell J. L. Morton, Mariam Awlia, Nadia Al‐Tamimi, Stephanie Saade, Yveline Pailles, Sónia Negrão, Mark Tester

**Affiliations:** ^1^ Division of Biological and Environmental Sciences and Engineering King Abdullah University of Science and Technology (KAUST) Thuwal 23955‐6900 Kingdom of Saudi Arabia

## Abstract

Salt stress limits the productivity of crops grown under saline conditions, leading to substantial losses of yield in saline soils and under brackish and saline irrigation. Salt tolerant crops could alleviate these losses while both increasing irrigation opportunities and reducing agricultural demands on dwindling freshwater resources. However, despite significant efforts, progress towards this goal has been limited, largely because of the genetic complexity of salt tolerance for agronomically important yield‐related traits. Consequently, the focus is shifting to the study of traits that contribute to overall tolerance, thus breaking down salt tolerance into components that are more genetically tractable. Greater consideration of the plasticity of salt tolerance mechanisms throughout development and across environmental conditions furthers this dissection. The demand for more sophisticated and comprehensive methodologies is being met by parallel advances in high‐throughput phenotyping and sequencing technologies that are enabling the multivariate characterisation of vast germplasm resources. Alongside steady improvements in statistical genetics models, forward genetics approaches for elucidating salt tolerance mechanisms are gaining momentum. Subsequent quantitative trait locus and gene validation has also become more accessible, most recently through advanced techniques in molecular biology and genomic analysis, facilitating the translation of findings to the field. Besides fuelling the improvement of established crop species, this progress also facilitates the domestication of naturally salt tolerant orphan crops. Taken together, these advances herald a promising era of discovery for research into the genetics of salt tolerance in plants.

## Significance Statement

The improvement of salt tolerance in crops is imperative for addressing yield penalties in saline soils and enabling the use of brackish water for irrigation. Major advances in high‐throughput phenotyping, data analysis, sequencing technologies and statistical genetics are empowering investigations into the mechanisms and genetics of salt tolerance, drawing upon an ever‐expanding suite of genetic resources. Bridging the genotype–phenotype gap to gain insights into the mechanisms of salt tolerance in plants is becoming practical.

## Introduction

Salt stress is a major constraint on plant performance. Saline soils alone, which account for one‐twentieth of the global total and one‐fifth of irrigated lands, have been estimated to incur $27.3 billion in agricultural damages annually (Qadir *et al*., 2014). This is a clear motivation for alleviating yield losses in saline soils, but the potential benefits from unlocking saline water (about 98% of global water resources) to expand irrigated agriculture and relieve growing pressure on freshwater resources is likely to be far greater. As such, addressing the issue of salt stress in plants offers a compelling contribution towards meeting the 50% increase in demand for food and freshwater required to sustain 10 billion people by 2050 (Gleeson *et al*., 2012; UN Water and Energy, 2014; Mekonnen and Hoekstra, 2016; FAO, 2017). Despite optimistic projections at the global scale, food scarcity will continue to persist regionally, particularly in sub‐Saharan Africa, the Middle East and South Asia where population growth is highest and agricultural outputs are most limited (FAO, 2017). In these most exigent regions, the attainable yield gap (i.e. the difference between observed yields for a given area and attainable yields under a similar climate) is substantial and constitutes a major challenge to present and future agricultural production. But given that 80% of the required increase in agricultural production by 2050 is expected to arise from yield increases (Alexandratos and Bruinsma, 2012) it also highlights an opportunity. For example, meeting attainable yields in rice, maize and wheat could increase production by 230%, 135% and 224%, respectively, in sub‐Saharan Africa and 129%, 83% and 61% in South Asia (Mueller *et al*., 2012). An increase in irrigation alone could provide yield gains of over 100% for these major crops, yet access to freshwater poses a serious limitation (Mueller *et al*., 2012; Famiglietti, 2014; Rodell *et al*., 2018). The development of crops with enhanced performance under saline conditions could enable the use of saline water sources, such as brackish water and partially desalinated or diluted seawater, for irrigation, and contribute to the sustainable intensification of agronomic systems under correct management.

Despite the potential of salt tolerant crops to significantly improve food production systems and the substantial efforts from the research community, relatively few salt tolerance genes or alleles have been identified and even fewer have led to real world applications. The genetic complexity of salt tolerance poses the greatest hurdle to progress. This review begins by revisiting how salt stress responses and salt tolerance are defined to provide a framework within which this complexity can be dissected to improve genetic resolution. We suggest that forward genetic approaches, genome‐wide association studies (GWAS) in particular, can provide unique insight ‘from the plant's perspective’ that can cut through this complexity and drive the discovery of new salt tolerance genes and alleles. We focus on the major challenges that have hindered such approaches in the past decade, alongside recent advances that are emerging as promising solutions and facilitators to uncover the genetic basis of salt tolerance.

## Salt Stress: Effects, Responses and Phenotypes in Plants

Salt stress can directly affect plant performance in several ways. High salinity causes a reduction in the osmotic and water potential of the growth medium, impeding water uptake. This, as well as the high levels of sodium ions themselves, can also affect nutrient absorption by disrupting native soil physico‐chemistry and the function of uptake mechanisms. Sodium ions are also able to enter the root through constitutively expressed ion channels and transporters, eventually spreading throughout the plant via the vascular system. Over time, sodium ions can accumulate to such levels as to incur cytotoxic effects or to cause osmotic imbalances within tissues, cells and subcellular compartments. Accumulation, and therefore damage, tends to be greater in aerial tissues because sodium ions are delivered there as solutes in the transpiration stream – as water is lost through transpiration, sodium ions are deposited cumulatively. These combined effects lead to the impairment of vital biological processes.

Plants can respond in various ways to these challenges. The responses of plants to salt stress are systemic in scope, occurring in both roots and shoots, at the organ, tissue, cellular and subcellular scales. Common responses include processes such as stress sensing and signalling, regulation of ion homeostasis, metabolism, stomatal aperture, photosynthesis, cell division and expansion, as well as changes in plant morphology and architecture, phenology and resource allocation (reviewed by Munns and Tester, 2008; Hairmansis *et al*., 2014). The responses employed, their nature, extent and the mechanisms that drive them can vary greatly between and within plant species. It is the interplay between the various direct effects of salt stress and the numerous potential plant responses that give rise to the wide diversity of phenotypic effects induced by salt stress that are observed across the plant kingdom.

Observable phenotypic effects of salt stress tend to be divided into rapid shoot ion‐independent effects (previously referred to as osmotic effects) and delayed ionic effects. Shoot ion‐independent effects typically include rapid reductions in turgor and stomatal closure and reduction in the rates of transpiration, photosynthesis and growth. Ionic effects develop gradually as sodium accumulation progresses, and generally involve a gradual decline in rates of photosynthesis, metabolism and growth, photosystem damage and early senescence.

It can be difficult to differentiate between the direct effects of salt stress and the plant's responses to them in establishing the salt stress phenotype. Plant responses *per se* could be viewed as those processes that lead to a deviation from the theoretical base effects of salt stress, rather than the observable phenotype as a whole. For example, a reduction in transpiration rates can be expected as a direct consequence of the reduced water potential of the growth medium, but it can also be a plant response that limits sodium accumulation. This response can be considered adaptive, but it bears noting that plant responses are not necessarily adaptive. For instance, deliberate, prolonged reduction of transpiration rates can prove to be a fatal plant response, as it usually implies reduced growth rates that in turn could jeopardise reproductive success. Depending on the context, it can be more advantageous to maintain high transpiration and growth rates, pushing through the reproductive cycle quickly to ensure seed production.

Investigation of the responses of plants to salt stress involves comparing genotypes with distinct phenotypes under salt stress, by studying either mutants or natural variation. Identifying the underlying genetics can remain highly challenging given the wide array of available plant responses that can give rise to a particular phenotypic effect. The issue is further exacerbated for phenotypic effects that relate to complex traits. To address this issue, the recommended strategy is to dissect the phenotypic effects of salt stress (e.g. effects on photosynthesis, effects on growth, effects on transpiration) and study plant responses and their genetic basis for each trait separately, as these are likely to arise from distinct mechanisms.

## Dissecting Plant Salt Tolerance into Contributing Traits and Mechanisms

In its broadest sense, salt tolerance is a measure of the maintenance of desirable plant performance traits under salt stress relative to control conditions. In practice, there is no single measure of salt tolerance, rather there are various indices by which maintenance of performance can be expressed, each having a slightly different focus (examples are presented in Table 1). For instance, the S/C index is the ratio of trait values under salt and control conditions, while the TOL index is the absolute difference between the two values. Generally, salt tolerance is measured using S/C index, due to its widespread use and broad relevance. There are also numerous plant performance traits that can be considered when evaluating salt tolerance. For application‐oriented research, salt tolerance should ultimately be assessed on the basis of economically or societally relevant traits in crops. Yield is typically the most important, but biomass, fruit or grain quality and others may be valid, depending on the species and market demands. Other traits (e.g. transpiration, photosynthetic activity, metabolite profiles) should be considered, not as measures of salt tolerance in themselves but rather as potential factors contributing to salt tolerance in terms of these traits of real‐world relevance.

**Table 1:** Examples of stress tolerance indices

Index	Described in
$\text{Salt tolerance index}:S/C=Y_{S}/Y_{C}$	Munns (2002)
$\text{Tolerance index}:\text{TOL}=Y_{C}-Y_{S}$	Fox and Rosielle (1982), Fernandez (1992)
$\text{Mean productivity index}:\text{MP}=\frac{Y_{C}+Y_{S}}{2}$	Rosielle and Hamblin (1981)
$\text{Geometric mean productivity}:\text{GMP}=\sqrt{\left(Y_{C}\times Y_{S}\right)}$	Fernandez (1992)
$\text{Stress susceptibility index}:\text{SSI}=\frac{\frac{Y_{C}-Y_{S}}{Y_{C}}}{\frac{Y_{\overset{¯}{C}}-Y_{\overset{¯}{S}}}{Y_{\overset{¯}{C}}}}$	Fischer and Maurer (1978)
$\text{Stress tolerance index}:\text{STI}=\frac{Y_{C}\times Y_{S}}{(Y_{\overset{¯}{C}}{)}^{2}}$	Fernandez (1992)
$\text{Stress weighted performance}:\text{SWP}=\frac{Y_{S}}{\sqrt{Y_{C}}}$	Saade *et al*. (2016)

*Y*_C_ and *Y*_S_ denote the value of the trait selected for assessing salt tolerance for a particular experimental unit, under control and stress conditions, respectively. $Y_{\overset{¯}{C}}$ and $Y_{\overset{¯}{S}}$ denote the population‐wide yield under control and stress conditions, respectively.

**Figure 1:**
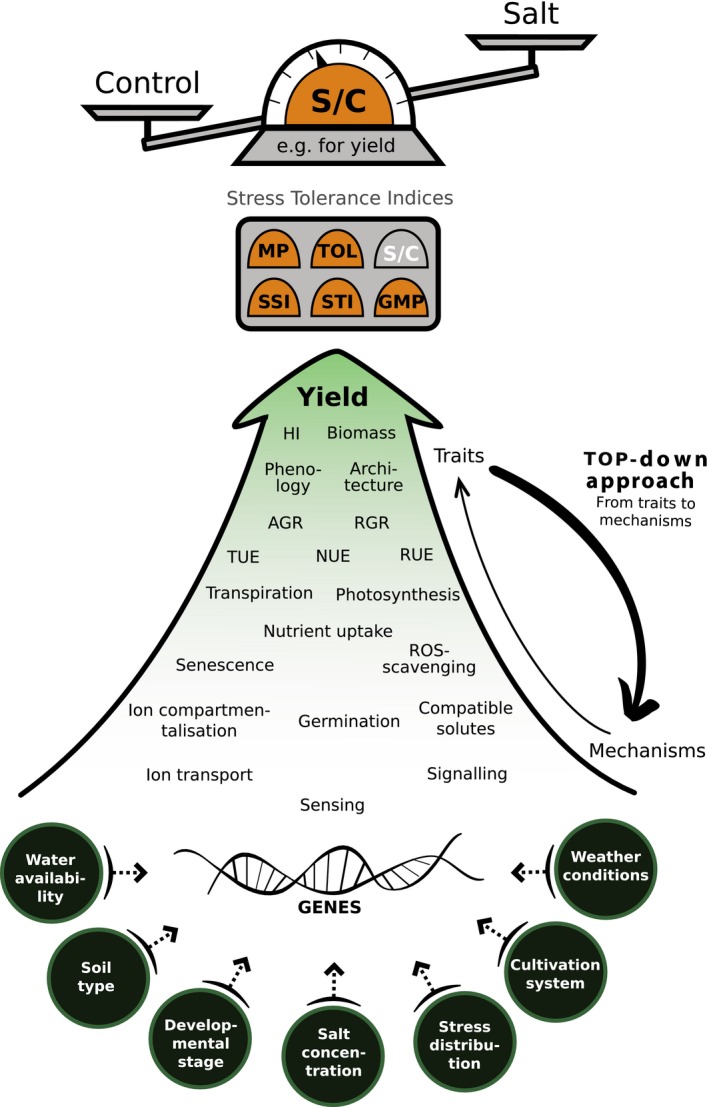
A plant scientist's guide to dissecting salt tolerance. For application‐oriented research, salt tolerance should be assessed according to the final trait of interest, most commonly yield – these are typically highly complex. Overall salt tolerance can be hierarchically dissected to identify downstream traits that are more genetically tractable. Traits that are seen to correlate with overall salt tolerance point towards processes that contribute to overall salt tolerance, i.e. salt tolerance mechanisms. A non‐exhaustive list of salt tolerance‐related traits and mechanisms is presented with a broad sense of their hierarchical organisation. This top‐down approach can eventually lead to the identification of underlying genetic components. Importantly, salt tolerance can be calculated using various stress tolerance indices, each of which provides a different perspective and a distinct focus on different aspects of salt tolerance. Moreover, any measure of tolerance can be influenced by the conditions under which it is assessed (examples are illustrated in circles) and the causal mechanisms underlying each measure can be distinct. HI, harvest index; RMR, root‐mass‐ratio; RGR, relative growth rate; TUE, NUE, RUE, transpiration‐, nutrient‐ and radiation‐use efficiency, respectively; S/C, salt tolerance index (Munns, 2002); TOL, tolerance index (Fox and Rosielle, 1982; Fernandez, 1992); MP, mean productivity index (Rosielle and Hamblin, 1981); GMP, geometric mean productivity (Fernandez, 1992); SSI, susceptibility index (Fischer and Maurer, 1978); STI, stress tolerance index (Fernandez, 1992).

Yield, for example, is a highly complex trait with many contributing factors. As a result of this complexity, it can be challenging to study the genetics of salt tolerance for yield *in toto*. Instead, it is more effective to study the genetics of the downstream traits that contribute to salt tolerance for yield. These contributing traits, their responses to salt stress and how they contribute to salt tolerance for yield can be dissected hierarchically (Figure 1). As such, salt tolerance for yield can arise from the maintenance of harvest index, i.e. the yield as fraction of overall biomass, which itself depends on maintained growth rates, in turn determined by traits such as transpiration, radiation and nutrient use efficiency (TUE, RUE, NUE, respectively), each of which are influenced by traits such as stomatal aperture and photosynthetic activity – and so on, down to the gene level. Each downstream component can contribute to salt tolerance for yield; thus constituting a salt tolerance mechanism. With a sufficiently comprehensive phenotyping methodology, insight into the eventual contribution of component traits to salt tolerance in terms of yield can be gained using correlation analyses. Although each of these components may remain complex themselves, this disambiguation helps to dissect salt tolerance for yield into constituent parts that are more genetically tractable than the whole.

The mechanisms that contribute to salt tolerance for yield, and subcomponents thereof, can vary between and within species, and also throughout development and across different environmental conditions. It is therefore important to account for factors such as genotype, salt stress concentration and distribution (e.g. temporal dynamics, topography), the developmental stage at which stress is imposed, weather conditions, cultivation practices, soil type and water availability (Greenway and Munns, 1980; Tal and Shannon, 1983; Mano and Takeda, 1997; Foolad, 1999; Yamaguchi and Blumwald, 2005; Munns and Tester, 2008; Negrão *et al*., 2017). For instance, salt tolerance in terms of relative growth rate can vary across the seedling, young and mature plant stages, with different tolerance mechanisms contributing significantly throughout development. The same can apply for different environmental conditions. Salt tolerance can therefore be further dissected and become more genetically tractable by refining experimental methodologies to account for these details. As such, measures of salt tolerance for yield under different conditions at different developmental stages, for example, can be considered in isolation; contributing traits and underlying genetics can then be determined independently. The same framework can be applied to dissect salt tolerance using any trait of interest.

The perspective is therefore shifting from viewing salt tolerance as a complex trait to a complex *of* traits that can be dissected through the use of different indices, traits and conditions. Moreover, implementing this deconstruction is becoming ever more practicable with the emergence of new phenotyping technologies and robust methodologies that allow the non‐destructive, simultaneous and high‐throughput measurement of numerous traits, including many that were previously intractable.

## Deploying Emerging Phenotyping Technologies for Comprehensive Salt Stress Phenotyping

Phenotyping the effects of salt stress in plants has traditionally been a relatively manual and laborious process. The focus has been on traits such as direct biomass measurements (e.g. shoot and root, fresh and dry), yield‐related traits (e.g. total seed mass, 1000‐grain weight, grain number), basic morphometric and architectural parameters (e.g. plant height, root length, number and length of side shoots, tillers, lateral roots etc.), visual scores of general plant health status (e.g. survival, senescence) and physiological measurements (e.g. ion contents, stomatal conductance, chlorophyll content), as well as laboratory‐based analyses of biochemical traits (e.g. metabolite and hormone levels) and gene expression (Campbell *et al*., 2017; Klem *et al*., 2017; Tschiersch *et al*., 2017; Ubbens *et al*., 2018). These methods have served well, particularly for reverse genetics approaches that typically deal with relatively small numbers of genotypes and thus sample sizes. However, for practical reasons, they have proven difficult to apply to forward genetics experiments that generally deal with large numbers of genotypes and sample sizes. Certainly, forward genetics salt tolerance screens have been widely performed on a staggering number of genotypes, yet these have relied on a very small number of very simple traits such as survival at the young seedling stage. The recent emergence of high‐throughput phenotyping (HTP) platforms is rapidly closing this gap, allowing the capture of large numbers of highly informative plant traits across vast experimental populations. Moreover, many of these new technologies are deployable in the field, offering much needed capabilities for studying key yield‐related traits that are necessary for application‐oriented research. Indeed, the vast majority of research into salt tolerance has thus far been extensively performed using model plants such as *Arabidopsis thaliana*, primarily due to practical constraints and the resulting focus of resources (methodological, genomic and genetic) towards this convenient organism. Yet, while Arabidopsis has without a doubt been the single most important species for the advancement of plant science over the past decades, its contributions to application‐oriented research into salt tolerance are somewhat constrained by its inherently low level of salt tolerance and lack of agronomically relevant yield‐related traits (Moller and Tester, 2007).

The past decade has seen the emergence of numerous HTP systems, which overwhelmingly rely on frequent, non‐invasive, automated imaging to phenotype tens, hundreds, even thousands of plants in a short time frame (Furbank and Tester, 2011; Ghanem *et al*., 2015; Junker *et al*., 2015). These systems facilitate the phenotyping of large numbers of genotypes in both controlled and field environments. The HTP platforms typically use red green blue (RGB), thermal infrared (TIR), chlorophyll fluorescence (ChlF) and, more recently, multispectral and hyperspectral imaging (see reviews by Humplik *et al*., 2015; Fahlgren *et al*., 2015). In RGB imaging, pixels are captured and subsequently analysed to inform on plant morphometric and colorimetric parameters, enabling the quantification of differences in growth rates or senescence, for example, under stress compared with control conditions (Rajendran *et al*., 2009; Al‐Tamimi *et al*., 2016; Awlia *et al*., 2016). Thermal infrared imaging is used to measure canopy and plant heat signatures to provide indirect assessments of stomatal conductance and transpiration, which negatively correlate with leaf temperature (Merlot *et al*., 2002; Sirault *et al*., 2009). Measuring the photosynthetic performance of plants directly by measuring steady‐state values upon light incidence or by observing the quenching kinetics of chlorophyll using ChIF imaging provides valuable insight into plant photosynthetic capacity and performance under stress (Baker, 2008; Jansen *et al*., 2009); (Campbell *et al*., 2015). Furthermore, multi‐ and hyperspectral imaging, which can capture narrow wavelength bands within and beyond the visible spectrum, show potential for the detection of stress responses (Fahlgren *et al*., 2015). Multi‐ and hyperspectral reflectance data are typically used to derive various vegetation indices that provide different estimations of the radiative properties of plant leaves or canopies. For example, the normalised difference vegetation index (NDVI) has been developed to assess plant health, while more complex spectral profiles afforded by hyperspectral imaging can be used to extrapolate the content of specific compounds, such as water, chlorophyll and even secondary metabolites like flavonoids and terpenoids (reviewed by Klem *et al*., 2017). The application of hyperspectral imaging for salt stress research is still in its infancy with only a few examples that focus on the estimation of vegetation indices as a proxy for crop stress (Naumann *et al*., 2009; Romer *et al*., 2012; Behmann *et al*., 2014; Sytar *et al*., 2017). Although hyperspectral imaging is more expensive than other imaging techniques and requires higher expertise for operation, image processing and data analysis and interpretation, it is expected to provide useful insights into the structural, biochemical and physiological traits related to salt stress responses. Recently, HTP methods have been widely employed to measure plants in the field by using these imaging tools with either ground‐based or aerial imaging to monitor the responses of crops to stress (Baluja *et al*., 2012; Grieder *et al*., 2015). Unmanned aerial vehicles (UAVs), such as drones and fixed‐wing aircraft, can measure plant traits at relatively high spatial and temporal resolutions. Moreover, they are becoming more affordable, accessible and common in crop research (reviewed by Araus and Cairns, 2014). However, data captured by UAVs require substantial computational power and highly trained personnel to analyse them, which poses challenges to the deployment of UAV‐based phenotyping platforms (Banan *et al*. 2018).

The major challenge of these imaging technologies, and HTP in general, is the massive number of data generated by these systems, which require sophisticated methods for data management, storage and analysis. To overcome this challenge, machine learning has become the leading method to facilitate data assimilation and trait identification for stress phenotyping (reviewed by Singh *et al*., 2016). New low‐cost and open‐source phenotyping technologies are continuously being developed, increasing their accessibility and usability for scientists and farmers alike (Tsaftaris and Noutsos, 2009; Fahlgren *et al*., 2015). For example, the recently developed PlantCV is a low‐cost HTP platform using low‐cost Raspberry Pi microcomputers and cameras to acquire plant image data together with open source analysis software (Gehan *et al*., 2017; Tovar *et al*., 2018).

As HTP systems can be employed for measuring large numbers of complex traits, they have great potential to measure the physiological effects of salt stress. For example, monitoring plant growth daily upon salt stress allows the distinction between tolerant and sensitive genotypes (Rajendran *et al*., 2009). The HTP protocols have also been described in Arabidopsis using RGB and ChlF imaging, which can identify a set of robust traits, such as growth rate, greenness and non‐photochemical quenching, for phenotyping the early responses to salt stress (Awlia *et al*., 2016). The use of HTP has allowed dissection of the genetic components of temporal salt stress responses in crops such as rice and barley (Hairmansis *et al*., 2014; Schilling *et al*., 2014; Campbell *et al*., 2015; Al‐Tamimi *et al*., 2016). The availability of reference genomes and genotypic datasets for several plant species has facilitated the association of genomic and phenotypic data using mapping methods to detect more quantitative trait loci (QTLs) that can significantly increase crop yield and stress tolerance in future breeding initiatives. The use of next‐generation phenotyping with new imaging technologies and novel traits is also paving the way for modelling genotype–phenotype interactions to reflect processes affecting crop growth and yield (Cobb *et al*., 2013). Moreover, the advances in monitoring environmental conditions, with the use of sensor technology, should be exploited to correctly characterise salt tolerance in terms of its conditional factors, for example soil salinity, water availability or climate (Figure 1). As such, genotype × environment (G×E) effects can be considered in the association and phenotypic modelling of traits contributing to salt tolerance.

As these new HTP platforms become increasingly sophisticated and accessible, our ability to screen large numbers of genotypes for forward genetics approaches grows steadily. Yet, to fully seize the opportunity of these new capabilities there is a clear need for expansive germplasm resources to characterise and mine the genetic components of salt tolerance. Fortuitously, the phenotyping revolution has been accompanied by another technological breakthrough that is unlocking vast genetic resources: the explosion of DNA sequencing technologies.

## Harnessing the Genetic Diversity of Exotic Germplasm

Current agricultural systems overwhelmingly rely on a small number of highly productive crops. A mere 20 plant species are used to fulfil 90% of the world's calorie requirements, with just three of these – rice, maize and wheat – supplying approximately 60% of the total (Massawe *et al*., 2016; Buchanan‐Wollaston *et al*., 2017). Domestication of these food crops from wild species began more than 10 000 years ago, over the course of which the performance and genetic architecture of the original progenitors were radically transformed (Tanksley and McCouch, 1997; McCouch, 2004). The selective breeding of a small number of wild varieties carrying beneficial traits, such as compact plant stature, non‐brittle rachis and loss of germination inhibition, produced landraces with superior performance but also gradually eroded the genetic diversity in successive populations (Dempewolf *et al*., 2017). The contraction of crop genetic diversity has been further exacerbated by modern plant breeding whereby high‐yielding elite cultivars are developed by crossing productive landraces while the wild ancestors with greater genetic variation but poor agronomic value are ignored (Gascuel *et al*., 2017). Such genetic bottlenecks have been confirmed experimentally (Haudry *et al*., 2007; Abbo *et al*., 2014). The inclination of farmers to switch from growing local varieties and landraces to genetically uniform and high‐yielding varieties has led to the loss of approximately 75% of genetic diversity in crops (Buchanan‐Wollaston *et al*., 2017). Importantly, as modern breeding tends to be carried out in optimal agricultural settings, the genetic components of disease and pest resistance, as well as tolerance to abiotic stresses such as salt stress, are often amongst the lost genetic fraction. As a result, attempts to identify genes conferring salt tolerance within commercial crop varieties have yielded limited results.

Several sources of genetic variation are available to complement the genetic paucity of modern elite cultivars. Genetic diversity generated artificially through mutagenesis has proven a valuable resource for various crop species, as variants can be directly produced in commercial germplasm (Caldwell *et al*., 2004; Mba, 2013; Nikam *et al*., 2015; Gulfishan *et al*., 2016; Çelik and Atak, 2017; Pando and Deza, 2017; Wang *et al*., 2017; Tu Anh *et al*., 2018). However, given that salt tolerance is most likely to arise from the concerted effects of numerous mechanisms, the potential to artificially create salt tolerant variants is surely limited. On the other hand, these are quite likely to emerge through natural selection in plants that are exposed to harsh environmental conditions, as observed in landraces or wild relatives of crops (Mayes *et al*., 2012). Therefore, it is essential to study the available naturally diverse germplasm. Global genebanks have collected approximately 2 million distinct plant accessions, of which a high percentage are landraces and wild relatives of crops (Commission on Genetic Resources for Food and Agriculture, 2010). The prospect of using such germplasm repositories as sources of natural variation for tolerance to environmental stresses such as salt tolerance has been widely discussed (Massawe *et al*., 2015; Tsujimoto *et al*., 2015; Hanin *et al*., 2016; Ali *et al*., 2017; Buchanan‐Wollaston *et al*., 2017; Dwivedi *et al*., 2017; Gascuel *et al*., 2017; Mabhaudhi *et al*., 2017; Zhang *et al*., 2017; Cheng, 2018). Assessments of genetic diversity have been performed for various crop germplasm collections, such as maize (Whitt *et al*., 2002; Liu *et al*., 2003; Patto *et al*., 2004; Laborda *et al*., 2005; Warburton *et al*., 2008; Prasanna, 2012; Zheng *et al*., 2013; Kuhn *et al*., 2014), wheat (Laido *et al*., 2013; Nielsen *et al*., 2014), rice (Cho *et al*., 2000; Temnykh *et al*., 2001; McCouch *et al*., 2002; Garris *et al*., 2005; Xu *et al*., 2012; Thomson *et al*., 2017), barley (Struss and Plieske, 1998; Parzies *et al*., 2000; Fernandez *et al*., 2002; Moragues *et al*., 2010) and tomato (Albrecht *et al*., 2010; Bauchet and Causse, 2012; Aflitos *et al*., 2014; Pailles *et al*., 2017). Furthermore, various studies using forward genetics approaches have already demonstrated the value of utilising diverse germplasm in practice by detecting loci associated with various measures of salt tolerance in established crops, for example in maize, rice, wheat, barley, soybean, rapeseed and alfalfa (Cui *et al*., 2015; Al‐Tamimi *et al*., 2016; Liu and Yu, 2017; Wan *et al*., 2017; Zeng *et al*., 2017; Oyiga *et al*., 2018).

**Figure 2:**
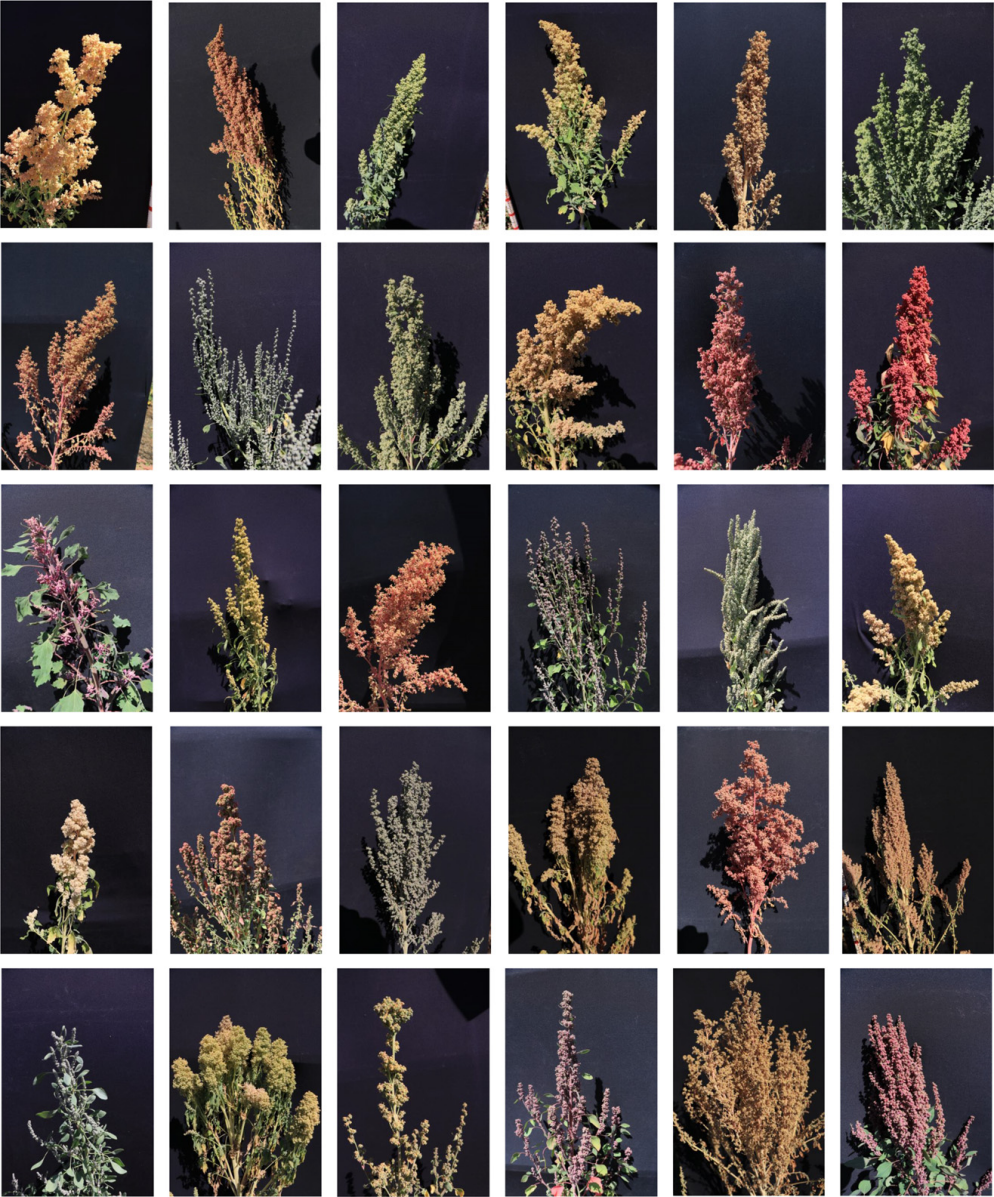
The phenotypic diversity of *Chenopodium quinoa* germplasm. The genetic diversity of quinoa germplasm can be clearly seen through the wide variety of panicle shapes, sizes and colours displayed in these field‐grown specimens (30 distinct accessions grown in the same season and field) (photo credits: Gabriele Fiene).

Overall, investigations into natural variation for salt tolerance remain scant considering the potential and the available germplasm resources. This is true for major crop species, but even more so for underutilised or orphan crops. Despite showing great potential in terms of salt tolerance, these valuable genetic resources have remained comparatively untapped. This is mainly due to the relative intractability of these germplasms. For instance, it can be challenging to rigorously phenotype highly diverse and undomesticated germplasm panels. Limited genetic and genomic resources for these germplasms as well as difficulties in effectively transferring beneficial alleles into elite varieties have also proved to be significant obstacles (Mayes *et al*., 2012; Wang *et al*., 2017). Nevertheless, the recent technological breakthroughs in the fields of phenotyping, DNA sequencing and molecular‐assisted breeding are increasingly facilitating the use of these genetic resources for crop improvement. For example, quinoa (*Chenopodium quinoa*), an undomesticated pseudo‐cereal that has gained popularity in developed countries due to its nutritional potential (Cheng, 2018), displays substantial genetic and phenotypic diversity (Figure 2), including accessions with high tolerance to salt stress (Jacobsen *et al*., 2003). The recent publication of two high‐quality quinoa genomes, alongside genomic data for a growing number of accessions and close relatives, provides great promise for detecting the genetic components underlying this species’ elevated salt tolerance (Jarvis *et al*., 2017; Zou *et al*., 2017). Interestingly, attempts are currently being made to domesticate these already salt tolerant orphan crops ahead of transferring this tolerance into major crops.

The accelerated development of DNA sequencing and genome assembly capabilities over the past decade is increasingly facilitating the genomic characterisation of new and uncharacterised germplasm – potential repositories of salt tolerance alleles and genes. Reference genome sequences are now available for over 300 plant species (as of March 2018; http://www.plabipd.de), with chromosome‐level assemblies for several major crops including rice, maize and wheat as well as less established species such as quinoa (Schnable, 2012; Mayer *et al*., 2014; Jackson, 2016; Jarvis *et al*., 2017). While the ever‐decreasing cost of short‐read sequencing platforms has driven much of this progress, it is the emerging long‐read, real‐time, linked‐read and single‐molecule sequencing techniques that are allowing researchers to overcome the intrinsic challenges of plant genomes, namely their large size, frequently complex ploidy levels and high repeat content (Bevan *et al*., 2017). The resulting high‐quality reference sequences provide the basis for genome analyses to identify structural variants responsible for species‐specific traits (e.g. the genes involved in fleshy fruit evolution in *Solanum lycopersicum*, or triterpenoid saponin biosynthesis in *C. quinoa*) (Sato *et al*., 2012; Jarvis *et al*., 2017). Such analyses have also provided insight into the potential genomic basis of drought and salt tolerance in the wild tomatoes *Solanum pennellii* and *Solanum pimpinellifolium*, respectively (Bolger *et al*., 2014)(Razali *et al*., 2018).

The greatest value of these genomic resources lies in their ability to empower the capture and characterisation of genetic variation. Indeed, high‐quality genomes beget high‐quality genotyping, from well‐established genotyping chips to more recent genotyping‐by‐sequencing methods, including various reduced representation sequencing and whole‐genome re‐sequencing approaches (Bevan *et al*., 2017; Dwivedi *et al*., 2017; Scheben *et al*., 2017). Using these approaches, vast numbers of accessions can now be genotyped relatively quickly and cheaply, with the potential to discover millions of single nucleotide polymorphisms (SNPs). Extensive genotypic resources are thus available for all sequenced major crop species, a number of wild relatives and increasingly for the aforementioned orphan crops, including diversity panels and mapping populations (e.g. bi‐parental, nested association) (reviewed by (Rasheed *et al*., 2018)). These growing resources will facilitate continued and improved investigations into the genetic basis of salt tolerance.

Beyond improvements in cost and throughput, increases in coverage, accuracy and the ability to discriminate haplotypes between homologous chromosomes offer growing insight into structural variations such as copy number variants (CNVs), presence–absence variants and chromosomal rearrangements (insertions, deletions, inversions, translocations) that are typically not captured by SNP‐based approaches. Our greater understanding of these structural variants, in particular CNVs that are widespread in plant genomes where they have been shown to contribute significantly to phenotypic variation, will provide much‐needed progress for the improvement of crop salt tolerance. Furthermore, the increased potential for *de novo* genome assembly for large numbers of accessions from a given species or closely related species facilitates the compilation of pangenomes, which summarise the structural variation observed across a large number of genotypes, providing a more comprehensive insight into structural variation (Golicz *et al*., 2016). Pangenomes have been compiled for many major crop species such as maize, rice, hexaploid wheat, soybean and *Brassica oleracea*, but have yet to be fully explored in terms of connecting genetic and phenotypic variation, particularly with regard to salt tolerance (Hirsch *et al*., 2014; Li *et al*., 2014; Golicz *et al*., 2016; Montenegro *et al*., 2017; Zhao *et al*., 2018).

The increasing availability of reference genomes and genotypic resources for exotic plant species, alongside the continuous development of tools to rigorously phenotype various components of salt tolerance, will enable the scrutiny of these exotic germplasm resources for salt tolerance, the underlying mechanisms and their genetic basis. Such applications will continue to rely heavily on existing and emerging statistical approaches that detect associations between genetic markers and phenotypic data through GWAS.

## Going Beyond Conventional Mapping

In forward genetic studies, the population size, phenotyping accuracy and linkage/association populations need to be carefully considered (Zhao *et al*., 2007). Striking a balance between the number of screened plant accessions, duration and temporal resolution time, traits to be measured and phenotyping precision are essential parameters to consider when planning an experimental setup that can reliably detect plausible loci with high statistical power. For marker‐trait association mapping, GWAS can provide insight into complex trait architecture, highlighting potential QTLs affecting traits that contribute to salt tolerance depending on the model used (Korte and Farlow, 2013). The basic and original approach in GWAS is to evaluate the association between each genotyped marker and a phenotype of interest that has been scored across a large number of individuals. The widely used conventional mixed linear model (MLM) association using the efficient mixed‐model association (EMMA) algorithm creates a linear mixed model that corrects for population structure using a marker‐based kinship matrix (Kang *et al*., 2008). In this section, available GWAS methods and models are summarised, presenting the advantages and disadvantages of each, for easy reference for plant scientists (Table 2).

**Table 2:** Comparison of available methods and models for genome‐wide association studies

Method	Description	Tools	Literature	Benefits	Limitations
Single locus method					
Exact methods					
EMMA	Efficient mixed model association	TASSEL (http://www.maizegenetics.net/tassel), EMMA R package	Kang *et al*. (2008)	Polygenic variance is re‐estimated with each marker analysed	Computationally intensive, only allows a single effect (samples or taxa) to be fit as a random effect. All other effects are treated as fixed
GEMMA	Genome‐wide efficient mixed model association	GEMMA software (http://stephenslab.uchicago.edu/software.html.)	Zhou and Stephens (2012)		
FaST‐LMM	Factored spectrally transformed linear mixed models	FaST‐LMM (python)	Lippert *et al*. (2011)		
Approximate methods					
EMMAX	Efficient mixed‐model association expedited	TASSEL	Kang *et al*. (2010)	Scales linearly with cohort size in both run time and memory use, substantially increase speed	Do not involve re‐estimating polygenic variance, less accuracy, systematic and appreciable underestimation of the most significant *P*‐values
CMLM	Compressed mixed linear model	TASSEL, GAPIT (http://zzlab.net/GAPIT)	Zhang *et al*. (2010)		
P3D	Population parameters previously determined	TASSEL	Zhang *et al*. (2010)		
Multi‐locus methods					
MLMM	Multi‐locus mixed model	Python(https://github.com/bvilhjal/mixmogam/), R (https://github.com/Gregor-Mendel-Institute/mlmm/)	Segura *et al*. (2012)	Identify evidence for allelic heterogeneity as well as interactions,unbiased analysis for interactions within the selected set of single nucleotide polymorphisms (SNPs), to some extent handle the confounding usually attributed to population structure, increased power, yielding reliable results for large datasets	Forward–backward inclusion of SNPs limits exploration of the huge model space
LMM‐Lasso	Least absolute shrinkage and selection operator	Python(https://github.com/BorgwardtLab/LMM-Lasso)	Rakitsch *et al*. (2013)		Computationally demanding
BSLMM	Bayesian sparse linear mixed model	GEMMA software (http://stephenslab.uchicago.edu/software.html)	Zhou *et al*. (2013)		
Multi‐trait methods					
MTMM	Multi‐trait mixed model	R software(https://github.com/Gregor-Mendel-Institute/mtmm)	Korte *et al*. (2012)	Considers both the within‐trait and between‐trait variance components	Difficult to implement in natural population‐based mapping owing to computational complexity; cannot control for population structure
Interaction model	Marker by treatment interaction model	Asreml R (http://datadryad.org/, doi:10.5061/dryad.3118j)	Al‐Tamimi *et al*. (2016)	Incorporates ‘main effects’ of the marker (SNP effect) and treatment as well as the marker‐by‐treatment interaction (SNP effect in response to the treatment	Cannot handle missing data
Bayesian methods					
SBL	Sparse Bayesian learning regression model	V2 SparseBayes software for Matlab^®^; SNPTEST, BIMBAM, SAS programs (EBAYES, SSVS and PENAL)	Tipping (2001)	Ability to estimate PVE, which is the total proportion of variance in response explained by relevant covariates, combine prior beliefs of marker effects, which are expressed in terms of prior distributions	Computationally intensive as it requires Markov chain Monte Carlo
One‐step GWAS					
ssGBLUP	Single‐step genomic best linear unbiased prediction	BLUPF90 software(http://nce.ads.uga.edu/software/)	Wang *et al*. (2012)	Greater power and precise estimate value	Computationally demanding, not implemented in plant species yet
One step interaction	One step marker by treatment interaction model	Asreml R		Greater power and precise estimate value	Computationally demanding; cannot work with missing data

There are a large number of software packages that facilitate essential GWAS analyses for estimating genotype–phenotype associations, but very few published comparisons of the available methods by which an informed selection can be made. The most commonly used packages for applications in plant science are TASSEL, GAPit and PLINK, with a growing number of studies being carried out using the R statistical computing software, which allows more flexibility in the development of complex genetic association models. However, no available software or R package has been able to provide the ideal balance between advanced statistical methods, time‐efficient simulations, accessibility and user‐friendliness. With the major developments in phenotypic technology and exploration in analytic approaches, GWAS has become an even more effective and efficient technique for studying the genetics underlying trait variation. Many improved statistical methods to increase statistical power, reduce false‐negative rates and incorporate biological context in GWAS results have recently been proposed in the field of plant genetics. Moreover, advanced forward genetics experimental setups, such as those that measure many traits over time for a large number of plants, are computationally demanding, adding to the requirement for more efficient methods for analysing high‐throughput phenotyping data. For this purpose, longitudinal genome‐wide association models have been developed to elucidate the genetic basis of the dynamic responses of rice growth to salt stress (Campbell *et al*., 2015). The model used accounts for both genetic relationships between genotypes due to the rice subpopulation structure and the inherent non‐independent nature of daily observations.

Mixed models and Bayesian approaches are being explored to take into account environmental and treatment effects, while other models account for the effects of genetic background, interactions between multiple phenotypes and confounding phenotypes. Studies have used phenotypic covariates to address relationships among traits. An alternative GWAS interaction model was developed which integrates control and saline conditions. This interaction model incorporates the ‘main effects’ of the marker (SNP effect) and treatment (control or salt) as well as the marker‐by‐treatment interaction (the SNP effect in response to the treatment). The use of this interaction model enabled the identification of significant loci specifically associated with salt stress (Al‐Tamimi *et al*., 2016). Another approach can be used to manage correlated phenotypes through deriving a fully parameterised multi‐trait mixed model (MTMM) that considers both the within‐trait and between‐trait variance components simultaneously for multiple traits. This GWAS approach was first used for an Arabidopsis dataset of flowering measurements in two different locations, identifying loci whose effects are specifically determined by the environment (Korte *et al*., 2012). A study in rice controlled for the confounding effects of flowering time on panicle architecture, by eliminating SNPs associated with the former, making it easier to investigate loci associated uniquely with panicle phenotypes and their effects on yield (Crowell *et al*., 2016). Such approaches can greatly improve the efficacy of GWAS approaches and their use is strongly encouraged for elucidating complex phenotypes such as salinity tolerance.

The conventional EMMA method is based on single‐locus tests (Kang *et al*., 2008), but as traits can be controlled by many loci with broader effects, as is the case for salt tolerance, this model does not produce a good estimate of marker effects. Numerous multi‐locus methodologies, which assume that several loci contribute to the phenotype of interest have been suggested, including penalised regressions (Hoggart *et al*., 2008; Yi and Xu, 2008; Wang *et al*., 2011; Waldmann *et al*., 2014) and even the elastic net (Waldmann *et al*., 2014). Another multi‐locus mixed model method involves the EMMA expedited (EMMAX) algorithm, which approximates the genetic and residual variance components and uses the same variance for all SNPs, re‐evaluating the genetic and residual variances at each step of the algorithm to avoid repetitive estimation of the variance component for each SNP (Segura *et al*., 2012). An example of a multi‐locus MLM used for salt tolerance traits in rice showed 20 quantitative trait nucleotides (QTNs) associated with 11 traits, including six QTNs affecting salt tolerance at the germination stage and 14 QTNs at the seedling stage (Naveed *et al*., 2018). Bayesian approaches have also extended to genetic association studies because of the practical and theoretical advantages shown in recent papers (Marchini *et al*., 2007; Servin and Stephens, 2007; Wakefield, 2007; Verzilli *et al*., 2008; Fridley, 2009). A number of software packages, for example SNPTEST (Marchini *et al*., 2007) and BIMBAM (Servin and Stephens, 2007) enable simple genome‐wide Bayesian analyses to be done easily and quickly on any standard desktop computer. Additionally, Wang *et al*. (2012) proposed new GWAS approaches called GWAS by single‐step genomic best linear unbiased prediction (ssGBLUP) and single‐step GWAS (ssGWAS). The ssGWAS method provides the most comprehensive information for genomic evaluation. The ssGWAS model allows all the available data, including genetic markers, phenotype records and pedigree information, to be examined simultaneously in one step. Many studies have recently validated this method and effectively implemented ssGWAS in pigs (Howard *et al*., 2015; Wu *et al*., 2018) and other species (Silva *et al*., 2017), achieving a greater power and more precise estimates than other models. The ssGWAS method is currently being tested in plant species by exploring the use of raw data (individual replicates) as opposed to standard approaches that use the means (across replicates), which leads to error inflation for the association analysis (unpublished data). Thus, genetic markers, the replicate measurements of each trait, spatial and design factors, and marker–treatment interaction can be simultaneously considered in a single model. Results from this approach are expected to increase in statistical power as each individual plant is considered as the input data instead of using the means of each genotype. This approach could be beneficial for complex quantitative salt tolerance traits that have various components that can be dissected effectively by a ssGWAS model, as we are able to detect significant loci specifically associated with salt stress due to the incorporation of the interaction model in the ssGWAS.

These rapidly evolving genome‐wide association models in crops are promising and highly useful tools for allele and marker discovery.

## Future Directions

To provide new insights into genotype–phenotype relations in terms of salt stress responses and tolerance in plants, the focus lies in the rapid advances of DNA sequencing technologies and HTP. This exciting era of omics tools promises to deliver powerful genetic and phenotypic resources that will enable a new generation of salt tolerant crops. The knowledge generated by these resources is expected to improve: (i) longitudinal GWAS combining HTP and new association methods (similar to what has been used in other research areas; Ning *et al*., 2017; Vujkovic *et al*., 2016); (ii) the genetic power to pinpoint the causal gene underlying a specific locus, for example, in Arabidopsis, the availability of 10 million SNPs has allowed the mapping of significant SNPs resulting from GWAS and specifically marking them by saturating the genome browser, thus uncovering the causal genes; and (iii) breeding programmes through identification of accessions carrying causal SNPs as donors for crossing schemes, and the use of causal SNPs to drive marker‐assisted selection. Moreover, speed breeding methods (Watson *et al*., 2018), which can achieve up to six generations per year instead of the typical two to three is expected to contribute to the fast delivery of salt tolerant crops.

Rapid advances in breeding strategies, together with the availability of new genomic tools have paved the way for the use of genomic selection (Crossa *et al*., 2017). Genomic selection uses a training population of individuals that have been phenotyped for the trait of interest and genotyped with genome‐wide markers that predict the breeding value of these individuals to enable the selection of superior lines and predict the performance of a genotype (Jannink *et al*., 2010). For example, different genomic models were tested to facilitate the selection of superior genotypes and candidate genes for breeding drought tolerance in subtropical maize germplasm (Shikha *et al*., 2017). The conjunction of genomic selection with the information provided by population structure, GWAS and the known genetic architecture of specific traits has successfully led to increased breeding efficiency in crops such as rice (Spindel *et al*., 2015). To our knowledge, genomic selection has not yet been used for breeding for salt tolerance in any plant species. However, we anticipate that the decreasing costs of genotyping and improved genomic models to predict breeding values will greatly contribute to accelerating the delivery of superior breeding lines with salt tolerance.

Moreover, the adoption of genome editing tools will enable the validation of causal gene function. For example, CRISPR‐Cas9 is expected to elucidate causal relationships between candidate genes identified by GWAS and phenotypic traits for the implementation of targeted molecular‐assisted breeding programmes (Yin *et al*., 2017). However, genome editing will not only assist the functional validation of loci of interest but will also enable the simultaneous modification of multiple genetic loci in elite crops, thus advancing breeding programmes and the translation of findings to the field.

Genetic diversity is a key asset for improving yield stability and stress tolerance. To improve agriculture, researchers use germplasm of crops and their wild relatives adapted to different environments as genetic resources (Tanksley and McCouch, 1997; Mickelbart *et al*., 2015). The use of wild relatives should not be confined to their use for increasing the genetic diversity of elite crops. One may consider the hypothesis of directly domesticating minor and orphan crop species with higher salt tolerance. Quinoa, for example, has a high potential for development as it exhibits a high tolerance to several abiotic stresses including salt tolerance (Jacobsen *et al*., 2003; Bazile *et al*., 2016; Jarvis *et al*., 2017), and thus breeding for important agronomic traits, such as shorter plants with fewer branches and more compact seed heads, will encourage commercial production in sub‐optimal environments where salinity is a major obstacle.

Here we have emphasised the importance of field HTP using UAVs. Quantitative trait locus mapping using traits collected by UAVs has enabled the identification of different alleles contributing to the rapid selection of high‐yielding varieties in rice (Tanger *et al*., 2017). We anticipate that machine learning algorithms will help leverage the outputs from emerging imaging technologies such as hyperspectral and thermal imaging to drive advances in the identification of genomic regions controlling the components of salt tolerance in important crop species.

Finally, it is worth highlighting that all the strategies discussed in this review can certainly be expanded beyond research into salt tolerance. For example, harnessing new genetic resources such as understudied crops or taking advantage of new imaging techniques using HTP along with new statistical association models will accelerate the discovery of new mechanisms of stress tolerance for any given stress. By establishing the connection between genotype and phenotype, we can advance the selection of high‐yield stress tolerant crops and improve agricultural production to address the growing pressures on food security.

## Conflict of interest

All authors declare that there are no conflicts of interest.

## References

[tpj14189-bib-0001] Abbo, S. , Pinhasi Van‐Oss, R. , Gopher, A. , Saranga, Y. , Ofner, I. and Peleg, Z. (2014) Plant domestication versus crop evolution: a conceptual framework for cereals and grain legumes. Trends Plant Sci. 19, 351–360. 24398119 10.1016/j.tplants.2013.12.002

[tpj14189-bib-0002] Aflitos, S. , Schijlen, E. , De Jong, H. ***et** **al*** (2014) Exploring genetic variation in the tomato (Solanum section Lycopersicon) clade by whole‐genome sequencing. Plant J. 80, 136–148. 25039268 10.1111/tpj.12616

[tpj14189-bib-0003] Albrecht, E. , Escobar, M. and Chetelat, R.T. (2010) Genetic diversity and population structure in the tomato‐like nightshades Solanum lycopersicoides and S. sitiens. Ann. Bot. 105, 535–554. 20154348 10.1093/aob/mcq009 PMC2850793

[tpj14189-bib-0004] Alexandratos, N. and Bruinsma, J. (2012) World agriculture towards 2030/2050: the 2012 revision. ESA Working paper No. 12‐03. Rome: FAO.

[tpj14189-bib-0005] Ali, J. , Xu, J.‐L. , Gao, Y.‐M. ***et** **al*** (2017) Harnessing the hidden genetic diversity for improving multiple abiotic stress tolerance in rice (Oryza sativa L.). PLoS ONE, 12, e0172515. 28278154 10.1371/journal.pone.0172515 PMC5344367

[tpj14189-bib-0006] Al‐Tamimi, N. , Brien, C. , Oakey, H. , Berger, B. , Saade, S. , Ho, Y.S. , Schmockel, S.M. , Tester, M. and Negrao, S. (2016) Salinity tolerance loci revealed in rice using high‐throughput non‐invasive phenotyping. Nat. Commun. 7, 13342. 27853175 10.1038/ncomms13342 PMC5118543

[tpj14189-bib-0007] Araus, J.L. and Cairns, J.E. (2014) Field high‐throughput phenotyping: the new crop breeding frontier. Trends Plant Sci. 19, 52–61. 24139902 10.1016/j.tplants.2013.09.008

[tpj14189-bib-0008] Awlia, M. , Nigro, A. , Faikus, J. , Schmoeckel, S.M. , Negrao, S. , Santelia, D. , Trtilek, M. , Tester, M. , Julkowska, M.M. and Panzarova, K. (2016) High‐throughput non‐destructive phenotyping of traits that contribute to salinity tolerance in Arabidopsis thaliana. Front. Plant Sci. 7, 1414. 27733855 10.3389/fpls.2016.01414 PMC5039194

[tpj14189-bib-0009] Baker, N.R. (2008) Chlorophyll fluorescence: a probe of photosynthesis in vivo. Annu. Rev. Plant Biol. 59, 89–113. 18444897 10.1146/annurev.arplant.59.032607.092759

[tpj14189-bib-0010] Baluja, J. , Diago, M.P. , Balda, P. , Zorer, R. , Meggio, F. , Morales, F. and Tardaguila, J. (2012) Assessment of vineyard water status variability by thermal and multispectral imagery using an unmanned aerial vehicle (UAV). Irrig. Sci. 30, 511–522.

[tpj14189-bib-0501] Banan, D. , Paul, R.E. , Feldman, M. , Holmes, M. , Schlake, H. , Baxter, I. , Jiang, H. and Leakey, A.D.B. (2018) High‐fidelity detection of crop biomass quantitative trait loci from low‐cost imaging in the field. Plant Direct, 2, 1–8. 10.1002/pld3.41 PMC6508524 31245708

[tpj14189-bib-0011] Bauchet, G. and Causse, M. (2012) Genetic diversity in tomato (*Solanum lycopersicum*) and its wild relatives In Genetic Diversity in Plants (Caliskan M., ed). Rijeka, Croatia: InTech, pp. 133–162.

[tpj14189-bib-0012] Bazile, D. , Jacobsen, S.‐E. and Verniau, A. (2016) The global expansion of quinoa: trends and limits. Front. Plant Sci. 7, 622. 27242826 10.3389/fpls.2016.00622 PMC4860459

[tpj14189-bib-0013] Behmann, J. , Steinrucken, J. and Plumer, L. (2014) Detection of early plant stress responses in hyperspectral images. ISPRS J. Photogramm. Remote Sens. 93, 98–111.

[tpj14189-bib-0014] Bevan, M.W. , Uauy, C. , Wulff, B.B.H. , Zhou, J. , Krasileva, K. and Clark, M.D. (2017) Genomic innovation for crop improvement. Nature, 543, 346–354. 28300107 10.1038/nature22011

[tpj14189-bib-0015] Bolger, A. , Scossa, F. , Bolger, M.E. ***et** **al*** (2014) The genome of the stress‐tolerant wild tomato species Solanum pennellii. Nat. Genet. 46, 1034–1038. 25064008 10.1038/ng.3046 PMC7036041

[tpj14189-bib-0016] Buchanan‐Wollaston, V. , Wilson, Z. , Tardieu, F. , Beynon, J. and Denby, K. (2017) Harnessing diversity from ecosystems to crops to genes. Food Energy Secur. 6, 19–25.

[tpj14189-bib-0017] Caldwell, D.G. , McCallum, N. , Shaw, P. , Muehlbauer, G.J. , Marshall, D.F. and Waugh, R. (2004) A structured mutant population for forward and reverse genetics in Barley (Hordeum vulgare L.). Plant J. 40, 143–150. 15361148 10.1111/j.1365-313X.2004.02190.x

[tpj14189-bib-0018] Campbell, M.T. , Knecht, A.C. , Berger, B. , Brien, C.J. , Wang, D. and Walia, H. (2015) Integrating image‐based phenomics and association analysis to dissect the genetic architecture of temporal salinity responses in rice. Plant Physiol. 168, 1476–U1697. 26111541 10.1104/pp.15.00450 PMC4528749

[tpj14189-bib-0019] Campbell, M.T. , Du, Q. , Liu, K. , Brien, C.J. , Berger, B. , Zhang, C. and Walia, H. (2017) A comprehensive image‐based phenomic analysis reveals the complex genetic architecture of shoot growth dynamics in rice (Oryza sativa). Plant Genome, 10, 10.3835/plantgenome2016.07.0064 28724075

[tpj14189-bib-0020] Çelik, Ö. and Atak, Ç. (2017) Applications of ionizing radiation in mutation breeding In New Insights on Gamma Rays (Maghraby A.M., ed). Rijeka, Croatia: InTech, pp. 111–132.

[tpj14189-bib-0021] Cheng, A. (2018) Review: shaping a sustainable food future by rediscovering long‐forgotten ancient grains. Plant Sci. 269, 136–142. 29606211 10.1016/j.plantsci.2018.01.018

[tpj14189-bib-0022] Cho, Y.G. , Ishii, T. , Temnykh, S. , Chen, X. , Lipovich, L. , McCouch, S.R. , Park, W.D. , Ayres, N. and Cartinhour, S. (2000) Diversity of microsatellites derived from genomic libraries and GenBank sequences in rice (Oryza sativa L.). Theor. Appl. Genet. 100, 713–722.

[tpj14189-bib-0023] Cobb, J.N. , Declerck, G. , Greenberg, A. , Clark, R. and McCouch, S. (2013) Next‐generation phenotyping: requirements and strategies for enhancing our understanding of genotype‐phenotype relationships and its relevance to crop improvement. Theor. Appl. Genet. 126, 867–887. 23471459 10.1007/s00122-013-2066-0 PMC3607725

[tpj14189-bib-0024] Crossa, J. , Perez‐Rodriguez, P. , Cuevas, J. ***et** **al*** (2017) Genomic selection in plant breeding: methods, models, and perspectives. Trends Plant Sci. 22, 961–975. 28965742 10.1016/j.tplants.2017.08.011

[tpj14189-bib-0025] Crowell, S. , Korniliev, P. , Falcao, A. , Ismail, A. , Gregorio, G. , Mezey, J. and McCouch, S. (2016) Genome‐wide association and high‐resolution phenotyping link Oryza sativa panicle traits to numerous trait‐specific QTL clusters. Nat. Commun. 7, 10527. 26841834 10.1038/ncomms10527 PMC4742901

[tpj14189-bib-0026] Cui, D.Z. , Wu, D.D. , Somarathna, Y. , Xu, C.Y. , Li, S. , Li, P. , Zhang, H. , Chen, H.B. and Zhao, L. (2015) QTL mapping for salt tolerance based on snp markers at the seedling stage in maize (Zea mays L.). Euphytica, 203, 273–283.

[tpj14189-bib-0027] Dempewolf, H. , Baute, G. , Anderson, J. , Kilian, B. , Smith, C. and Guarino, L. (2017) Past and future use of wild relatives in crop breeding. Crop Sci. 57, 1070.

[tpj14189-bib-0028] Dwivedi, S.L. , Scheben, A. , Edwards, D. , Spillane, C. and Ortiz, R. (2017) Assessing and exploiting functional diversity in germplasm pools to enhance abiotic stress adaptation and yield in cereals and food legumes. Front. Plant Sci. 8, 1461. 28900432 10.3389/fpls.2017.01461 PMC5581882

[tpj14189-bib-0029] Fahlgren, N. , Gehan, M.A. and Baxter, I. (2015) Lights, camera, action: high‐throughput plant phenotyping is ready for a close‐up. Curr. Opin. Plant Biol. 24, 93–99. 25733069 10.1016/j.pbi.2015.02.006

[tpj14189-bib-0030] Famiglietti, J.S. (2014) The global groundwater crisis. Nat. Clim. Chang. 4, 945–948.

[tpj14189-bib-0031] FAO . (2017) The Future of Food and Agriculture ‐ Trends and Challenges. Rome: Food and Agriculture Organization of the United Nations.

[tpj14189-bib-0032] Fernandez, G.C.J. (1992) Effective selection criteria for assessing stress tolerance. In *Proceedings of the international symposium on adaptation of vegetable and other food crops in temperature and water stress* (Kuo, C.G., Ed)., 1992 Tainan: AVRDC Publication, pp. 257–270.

[tpj14189-bib-0033] Fernandez, M.E. , Figueiras, A.M. and Benito, C. (2002) The use of ISSR and RAPD markers for detecting DNA polymorphism, genotype identification and genetic diversity among barley cultivars with known origin. Theor. Appl. Genet. 104, 845–851. 12582645 10.1007/s00122-001-0848-2

[tpj14189-bib-0034] Fischer, R.A. and Maurer, R. (1978) Drought resistance in spring wheat cultivars. 1. Grain‐yield responses. Aust. J. Agric. Res. 29, 897–912.

[tpj14189-bib-0035] Foolad, M.R. (1999) Comparison of salt tolerance during seed germination and vegetative growth in tomato by QTL mapping. Genome, 42, 727–734.

[tpj14189-bib-0036] Fox, P.N. and Rosielle, A.A. (1982) Reducing the influence of environmental main‐effects on pattern‐analysis of plant‐breeding environments. Euphytica, 31, 645–656.

[tpj14189-bib-0037] Fridley, B.L. (2009) Bayesian variable and model selection methods for genetic association studies. Genet. Epidemiol. 33, 27–37. 18618760 10.1002/gepi.20353

[tpj14189-bib-0038] Furbank, R.T. and Tester, M. (2011) Phenomics ‐ technologies to relieve the phenotyping bottleneck. Trends Plant Sci. 16, 635–644. 22074787 10.1016/j.tplants.2011.09.005

[tpj14189-bib-0039] Garris, A.J. , Tai, T.H. , Coburn, J. , Kresovich, S. and McCouch, S. (2005) Genetic structure and diversity in Oryza sativa L. Genetics, 169, 1631–1638. 15654106 10.1534/genetics.104.035642 PMC1449546

[tpj14189-bib-0040] Gascuel, Q. , Diretto, G. , Monforte, A.J. , Fortes, A.M. and Granell, A. (2017) Use of natural diversity and biotechnology to increase the quality and nutritional content of tomato and grape. Front. Plant Sci. 8, 652. 28553296 10.3389/fpls.2017.00652 PMC5427129

[tpj14189-bib-0041] Gehan, M.A. , Fahlgren, N. , Abbasi, A. ***et** **al*** (2017) PlantCV v2: image analysis software for high‐throughput plant phenotyping. PeerJ, 5, e4088. 29209576 10.7717/peerj.4088 PMC5713628

[tpj14189-bib-0042] Ghanem, M.E. , Marrou, H. and Sinclair, T.R. (2015) Physiological phenotyping of plants for crop improvement. Trends Plant Sci. 20, 139–144. 25524213 10.1016/j.tplants.2014.11.006

[tpj14189-bib-0043] Gleeson, T. , Wada, Y. , Bierkens, M.F. and van Beek, L.P. (2012) Water balance of global aquifers revealed by groundwater footprint. Nature, 488, 197–200. 22874965 10.1038/nature11295

[tpj14189-bib-0044] Golicz, A.A. , Bayer, P.E. , Barker, G.C. ***et** **al*** (2016) The pangenome of an agronomically important crop plant Brassica oleracea. Nat. Commun. 7, 13390. 27834372 10.1038/ncomms13390 PMC5114598

[tpj14189-bib-0045] Greenway, H. and Munns, R. (1980) Mechanisms of salt tolerance in non‐halophytes. Annu. Rev. Plant Physiol. Plant Mol. Biol. 31, 149–190.

[tpj14189-bib-0046] Grieder, C. , Hund, A. and Walter, A. (2015) Image based phenotyping during winter: a powerful tool to assess wheat genetic variation in growth response to temperature. Funct. Plant Biol. 42, 387–396. 10.1071/FP14226 32480683

[tpj14189-bib-0047] Gulfishan, M. , Bhat, T.A. and Oves, M. (2016). Mutants as a genetic resource for future crop improvement In Advances in Plant Breeding Strategies: Breeding, Biotechnology and Molecular Tools (Al‐Khayri J.M., Jain S.M. and Johnson D.V., eds). Cham: Springer International Publishing, pp. 95–112.

[tpj14189-bib-0048] Hairmansis, A. , Berger, B. , Tester, M. and Roy, S.J. (2014) Image‐based phenotyping for non‐destructive screening of different salinity tolerance traits in rice. Rice, 7, 16. 26055997 10.1186/s12284-014-0016-3 PMC4884049

[tpj14189-bib-0049] Hanin, M. , Ebel, C. , Ngom, M. , Laplaze, L. and Masmoudi, K. (2016) New insights on plant salt tolerance mechanisms and their potential use for breeding. Front. Plant Sci. 7, 1787. 27965692 10.3389/fpls.2016.01787 PMC5126725

[tpj14189-bib-0050] Haudry, A. , Cenci, A. , Ravel, C. ***et** **al*** (2007) Grinding up wheat: a massive loss of nucleotide diversity since domestication. Mol. Biol. Evol. 24, 1506–1517. 17443011 10.1093/molbev/msm077

[tpj14189-bib-0051] Hirsch, C.N. , Foerster, J.M. , Johnson, J.M. ***et** **al*** (2014) Insights into the Maize Pan‐Genome and Pan‐Transcriptome. Plant Cell, 26, 121–135. 24488960 10.1105/tpc.113.119982 PMC3963563

[tpj14189-bib-0052] Hoggart, C.J. , Whittaker, J.C. , De Iorio, M. and Balding, D.J. (2008) Simultaneous analysis of All SNPs in genome‐wide and re‐sequencing association studies. PLoS Genet. 4, e1000130. 18654633 10.1371/journal.pgen.1000130 PMC2464715

[tpj14189-bib-0053] Howard, J.T. , Jiao, S. , Tiezzi, F. , Huang, Y. , Gray, K.A. and Maltecca, C. (2015) Genome‐wide association study on legendre random regression coefficients for the growth and feed intake trajectory on Duroc Boars. BMC Genet. 16, 59. 26024912 10.1186/s12863-015-0218-8 PMC4449572

[tpj14189-bib-0054] Humplik, J.F. , Lazar, D. , Husickova, A. and Spichal, L. (2015) Automated phenotyping of plant shoots using imaging methods for analysis of plant stress responses ‐ a review. Plant Methods, 11, 29. 25904970 10.1186/s13007-015-0072-8 PMC4406171

[tpj14189-bib-0055] Jackson, S.A. (2016) Rice: the first crop genome. Rice, 9, 14. 27003180 10.1186/s12284-016-0087-4 PMC4803718

[tpj14189-bib-0056] Jacobsen, S.‐E. , Mujica, A. and Jensen, C.R. (2003) The resistance of Quinoa (Chenopodium quinoa Willd.) to adverse abiotic factors. Food Rev. Int. 19, 8755–9129.

[tpj14189-bib-0057] Jannink, J.L. , Lorenz, A.J. and Iwata, H. (2010) Genomic selection in plant breeding: from theory to practice. Brief Funct. Genomics, 9, 166–177. 20156985 10.1093/bfgp/elq001

[tpj14189-bib-0058] Jansen, M. , Gilmer, F. , Biskup, B. ***et** **al*** (2009) Simultaneous phenotyping of leaf growth and chlorophyll fluorescence via GROWSCREEN FLUORO allows detection of stress tolerance in Arabidopsis thaliana and other rosette plants. Funct. Plant Biol. 36, 902–914. 10.1071/FP09095 32688701

[tpj14189-bib-0059] Jarvis, D.E. , Ho, Y.S. , Lightfoot, D.J. ***et** **al*** (2017) The genome of *Chenopodium quinoa* . Nature, 542, 307. 28178233 10.1038/nature21370

[tpj14189-bib-0060] Junker, A. , Muraya, M.M. , Weigelt‐Fischer, K. , Arana‐Ceballos, F. , Klukas, C. , Melchinger, A.E. , Meyer, R.C. , Riewe, D. and Altmann, T. (2015) Optimizing experimental procedures for quantitative evaluation of crop plant performance in high throughput phenotyping systems. Front. Plant Sci. 5, 770. 25653655 10.3389/fpls.2014.00770 PMC4299434

[tpj14189-bib-0061] Kang, H.M. , Zaitlen, N.A. , Wade, C.M. , Kirby, A. , Heckerman, D. , Daly, M.J. and Eskin, E. (2008) Efficient control of population structure in model organism association mapping. Genetics, 178, 1709–1723. 18385116 10.1534/genetics.107.080101 PMC2278096

[tpj14189-bib-0062] Kang, H.M. , Sul, J.H. , Service, S.K. , Zaitlen, N.A. , Kong, S. , Freimer, N.B. , Sabatti, C. and Eskin, E. (2010) Variance component model to account for sample structure in genome‐wide association studies. Nat. Genet. 42, 348–354. 20208533 10.1038/ng.548 PMC3092069

[tpj14189-bib-0063] Klem, K. , Mishra, K.B. , Novotna, K. , Rapantova, B. , Hodanova, P. , Mishra, A. , Kovac, D. and Urban, O. (2017) Distinct growth and physiological responses of Arabidopsis thaliana natural accessions to drought stress and their detection using spectral reflectance and thermal imaging. Funct. Plant Biol. 44, 312–323. 10.1071/FP16194 32480566

[tpj14189-bib-0064] Korte, A. and Farlow, A. (2013) The advantages and limitations of trait analysis with GWAS: a review. Plant Methods, 9, 29. 23876160 10.1186/1746-4811-9-29 PMC3750305

[tpj14189-bib-0065] Korte, A. , Vilhjalmsson, B.J. , Segura, V. , Platt, A. , Long, Q. and Nordborg, M. (2012) A mixed‐model approach for genome‐wide association studies of correlated traits in structured populations. Nat. Genet. 44, 1066–1071. 22902788 10.1038/ng.2376 PMC3432668

[tpj14189-bib-0066] Kuhn, B.C. , López‐Ribera, I. , Da Silva Machado, M.D.F.P. and Vicient, C.M. (2014) Genetic diversity of maize germplasm assessed by retrotransposon‐based markers. Electrophoresis, 35, 1921–1927. 24634146 10.1002/elps.201400038

[tpj14189-bib-0067] Laborda, P.R. , Oliveira, K.M. , Garcia, A.A.F. , Paterniani, M.E.A.G.Z. and De Souza, A.P. (2005) Tropical maize germplasm: what can we say about its genetic diversity in the light of molecular markers? Theor. Appl. Genet. 111, 1288–1299. 16133309 10.1007/s00122-005-0055-7

[tpj14189-bib-0068] Laido, G. , Mangini, G. , Taranto, F. , Gadaleta, A. , Blanco, A. , Cattivelli, L. , Marone, D. , Mastrangelo, A.M. , Papa, R. and De Vita, P. (2013) Genetic diversity and population structure of tetraploid wheats (Triticum turgidum L.) estimated by SSR, DArT and pedigree data. PLoS ONE, 8, e67280. 23826256 10.1371/journal.pone.0067280 PMC3694930

[tpj14189-bib-0069] Li, Y.H. , Zhou, G. , Ma, J. ***et** **al*** (2014) De novo assembly of soybean wild relatives for pan‐genome analysis of diversity and agronomic traits. Nat. Biotechnol. 32, 1045–1052. 25218520 10.1038/nbt.2979

[tpj14189-bib-0070] Lippert, C. , Listgarten, J. , Liu, Y. , Kadie, C.M. , Davidson, R.I. and Heckerman, D. (2011) FaST linear mixed models for genome‐wide association studies. Nat. Methods, 8, 833–835. 21892150 10.1038/nmeth.1681

[tpj14189-bib-0071] Liu, X.P. and Yu, L.X. (2017) Genome‐wide association mapping of loci associated with plant growth and forage production under salt stress in Alfalfa (Medicago sativa L.). Front. Plant Sci. 8, 853. 28596776 10.3389/fpls.2017.00853 PMC5442208

[tpj14189-bib-0072] Liu, K. , Goodman, M. , Muse, S. , Smith, J.S. , Buckler, E. and Doebley, J. (2003) Genetic structure and diversity among maize inbred lines as inferred from DNA microsatellites. Genetics, 165, 2117–2128. 14704191 10.1093/genetics/165.4.2117 PMC1462894

[tpj14189-bib-0073] Mabhaudhi, T. , Chimonyo, V.G.P. , Chibarabada, T.P. and Modi, A.T. (2017) Developing a roadmap for improving neglected and underutilized crops: a case study of South Africa. Front. Plant Sci. 8, 2143. 29312397 10.3389/fpls.2017.02143 PMC5735103

[tpj14189-bib-0074] Mano, Y. and Takeda, K. (1997) Mapping quantitative trait loci for salt tolerance at germination and the seedling stage in barley (Hordeum vulgare L). Euphytica, 94, 263–272.

[tpj14189-bib-0075] Marchini, J. , Howie, B. , Myers, S. , McVean, G. and Donnelly, P. (2007) A new multipoint method for genome‐wide association studies by imputation of genotypes. Nat. Genet. 39, 906. 17572673 10.1038/ng2088

[tpj14189-bib-0076] Massawe, F.J. , Mayes, S. , Cheng, A. ***et** **al*** (2015) The potential for underutilised crops to improve food security in the face of climate change. Procedia Environ. Sci. 29, 140–141.

[tpj14189-bib-0077] Massawe, F. , Mayes, S. and Cheng, A. (2016) Crop diversity: an unexploited treasure trove for food security. Trends Plant Sci. 21, 365–368. 27131298 10.1016/j.tplants.2016.02.006

[tpj14189-bib-0078] Mayer, K.F.X. , Rogers, J. , Dolezel, J. ***et** **al*** (2014) A chromosome‐based draft sequence of the hexaploid bread wheat (Triticum aestivum) genome. Science, 345, 1251788. 25035500 10.1126/science.1251788

[tpj14189-bib-0079] Mayes, S. , Massawe, F.J. , Alderson, P.G. , Roberts, J.A. , Azam‐Ali, S.N. and Hermann, M. (2012) The potential for underutilized crops to improve security of food production. J. Exp. Bot. 63, 1075–1079. 22131158 10.1093/jxb/err396

[tpj14189-bib-0080] Mba, C. (2013) Induced mutations unleash the potentials of plant genetic resources for food and agriculture. Agronomy, 3, 200–231.

[tpj14189-bib-0081] McCouch, S. (2004) Diversifying selection in plant breeding. PLoS Biol. 2, e347. 15486582 10.1371/journal.pbio.0020347 PMC521731

[tpj14189-bib-0082] McCouch, S.R. , Teytelman, L. , Xu, Y.B. ***et** **al*** (2002) Development and mapping of 2240 new SSR markers for rice (Oryza sativa L.). DNA Res. 9, 199–207. 12597276 10.1093/dnares/9.6.199

[tpj14189-bib-0083] Mekonnen, M.M. and Hoekstra, A.Y. (2016) Four billion people facing severe water scarcity. Sci. Adv. 2, e1500323. 26933676 10.1126/sciadv.1500323 PMC4758739

[tpj14189-bib-0084] Merlot, S. , Mustilli, A.C. , Genty, B. , North, H. , Lefebvre, V. , Sotta, B. , Vavasseur, A. and Giraudat, J. (2002) Use of infrared thermal imaging to isolate Arabidopsis mutants defective in stomatal regulation. Plant J. 30, 601–609. 12047634 10.1046/j.1365-313x.2002.01322.x

[tpj14189-bib-0085] Mickelbart, M.V. , Hasegawa, P.M. and Bailey‐Serres, J. (2015) Genetic mechanisms of abiotic stress tolerance that translate to crop yield stability. Nat. Rev. Genet. 16, 237–251. 25752530 10.1038/nrg3901

[tpj14189-bib-0086] Moller, I.S. and Tester, M. (2007) Salinity tolerance of Arabidopsis: a good model for cereals? Trends Plant Sci. 12, 534–540. 18023242 10.1016/j.tplants.2007.09.009

[tpj14189-bib-0087] Montenegro, J.D. , Golicz, A.A. , Bayer, P.E. ***et** **al*** (2017) The pangenome of hexaploid bread wheat. Plant J. 90, 1007–1013. 28231383 10.1111/tpj.13515

[tpj14189-bib-0088] Moragues, M. , Comadran, J. , Waugh, R. , Milne, I. , Flavell, A.J. and Russell, J.R. (2010) Effects of ascertainment bias and marker number on estimations of barley diversity from high‐throughput SNP genotype data. Theor. Appl. Genet. 120, 1525–1534. 20157694 10.1007/s00122-010-1273-1

[tpj14189-bib-0089] Mueller, N.D. , Gerber, J.S. , Johnston, M. , Ray, D.K. , Ramankutty, N. and Foley, J.A. (2012) Closing yield gaps through nutrient and water management. Nature, 490, 254–257. 22932270 10.1038/nature11420

[tpj14189-bib-0090] Munns, R. (2002) Comparative physiology of salt and water stress. Plant Cell Environ. 25, 239–250. 11841667 10.1046/j.0016-8025.2001.00808.x

[tpj14189-bib-0091] Munns, R. and Tester, M. (2008) Mechanisms of salinity tolerance. Annu. Rev. Plant Biol. 59, 651–681. 18444910 10.1146/annurev.arplant.59.032607.092911

[tpj14189-bib-0092] Naumann, J.C. , Young, D.R. and Anderson, J.E. (2009) Spatial variations in salinity stress across a coastal landscape using vegetation indices derived from hyperspectral imagery. Plant Ecol. 202, 285–297.

[tpj14189-bib-0093] Naveed, S.A. , Zhang, F. , Zhang, J. , Zheng, T.Q. , Meng, L.J. , Pang, Y.L. , Xu, J.L. and Li, Z.K. (2018) Identification of QTN and candidate genes for salinity tolerance at the germination and seedling stages in rice by genome‐wide association analyses. Sci. Rep. 8, 6505. 29695843 10.1038/s41598-018-24946-3 PMC5916932

[tpj14189-bib-0094] Negrão, S. , Schmöckel, S.M. and Tester, M. (2017) Evaluating physiological responses of plants to salinity stress. Ann. Bot. 119, 1–11. 27707746 10.1093/aob/mcw191 PMC5218372

[tpj14189-bib-0095] Nielsen, N.H. , Backes, G. , Stougaard, J. , Andersen, S.U. and Jahoor, A. (2014) Genetic diversity and population structure analysis of european hexaploid bread wheat (Triticum aestivum L.) varieties. PLoS ONE, 9, e94000. 24718292 10.1371/journal.pone.0094000 PMC3981729

[tpj14189-bib-0096] Nikam, A.A. , Devarumath, R.M. , Ahuja, A. , Babu, H. , Shitole, M.G. and Suprasanna, P. (2015) Radiation‐induced in vitro mutagenesis system for salt tolerance and other agronomic characters in sugarcane (Saccharum officinarum L.). Crop J. 3, 46–56.

[tpj14189-bib-0097] Ning, C. , Kang, H. , Zhou, L. , Wang, D. , Wang, H. , Wang, A. , Fu, J. , Zhang, S. and Liu, J. (2017) Performance gains in genome‐wide association studies for longitudinal traits via modeling time‐varied effects. Sci. Rep. 7, 590. 28377602 10.1038/s41598-017-00638-2 PMC5428860

[tpj14189-bib-0098] Oyiga, B.C. , Sharma, R.C. , Baum, M. , Ogbonnaya, F.C. , Leon, J. and Ballvora, A. (2018) Allelic variations and differential expressions detected at quantitative trait loci for salt stress tolerance in wheat. Plant Cell Environ. 41, 919–935. 28044314 10.1111/pce.12898

[tpj14189-bib-0099] Pailles, Y. , Ho, S. , Pires, I.S. , Tester, M. , Negrão, S. and Schmöckel, S.M. (2017) Genetic diversity and population structure of two tomato species from the Galapagos Islands. Front. Plant Sci. 8, 1–11. 28261227 10.3389/fpls.2017.00138 PMC5309213

[tpj14189-bib-0100] Pando, G.L. and Deza, P. (2017) Development of advanced mutant lines of native grains through radiation‐induced Mutagenesis in Peru. Hortic. Int. J. 1, 15–19.

[tpj14189-bib-0101] Parzies, H.K. , Spoor, W. and Ennos, R.A. (2000) Genetic diversity of barley landrace accessions (Hordeum vulgare ssp vulgare) conserved for different lengths of time in ex situ gene banks. Heredity, 84, 476–486. 10849072 10.1046/j.1365-2540.2000.00705.x

[tpj14189-bib-0102] Patto, M.C.V. , Satovic, Z. , Pêgo, S. and Fevereiro, P. (2004) Assessing the genetic diversity of Portuguese maize germplasm using microsatellite markers. Euphytica, 137, 63–72.

[tpj14189-bib-0103] Prasanna, B.M. (2012) Diversity in global maize germplasm: characterization and utilization. J. Biosci. 37, 843–855. 23107920 10.1007/s12038-012-9227-1

[tpj14189-bib-0104] Qadir, M. , Quillerou, E. , Nangia, V. , Murtaza, G. , Singh, M. , Thomas, R.J. , Drechsel, P. and Noble, A.D. (2014) Economics of salt‐induced land degradation and restoration. Nat. Resour. Forum, 38, 282–295.

[tpj14189-bib-0105] Rajendran, K. , Tester, M. and Roy, S.J. (2009) Quantifying the three main components of salinity tolerance in cereals. Plant Cell Environ. 32, 237–249. 19054352 10.1111/j.1365-3040.2008.01916.x

[tpj14189-bib-0106] Rakitsch, B. , Lippert, C. , Stegle, O. and Borgwardt, K. (2013) A Lasso multi‐marker mixed model for association mapping with population structure correction. Bioinformatics, 29, 206–214. 23175758 10.1093/bioinformatics/bts669

[tpj14189-bib-0107] Rasheed, A. , Mujeeb‐Kazi, A. , Ogbonnaya, F.C. , He, Z. and Rajaram, S. (2018) Wheat genetic resources in the post‐genomics era: promise and challenges. Ann. Bot. 121, 603–616. 29240874 10.1093/aob/mcx148 PMC5852999

[tpj14189-bib-0108] Razali, R. , Bougouffa, S. , Morton, M.J.L. ***et** **al*** (2018) The genome sequence of the wild tomato *Solanum pimpinellifolium* provides insights into salinity tolerance. Front. Plant Sci. 9, 1402. 30349549 10.3389/fpls.2018.01402 PMC6186997

[tpj14189-bib-0109] Rodell, M. , Famiglietti, J.S. , Wiese, D.N. , Reager, J.T. , Beaudoing, H.K. , Landerer, F.W. and Lo, M.H. (2018) Emerging trends in global freshwater availability. Nature, 557, 651–659. 29769728 10.1038/s41586-018-0123-1 PMC6077847

[tpj14189-bib-0110] Romer, C. , Wahabzada, M. , Ballvora, A. ***et** **al*** (2012) Early drought stress detection in cereals: simplex volume maximisation for hyperspectral image analysis. Funct. Plant Biol. 39, 878–890. 10.1071/FP12060 32480838

[tpj14189-bib-0111] Rosielle, A.A. and Hamblin, J. (1981) Theoretical aspects of selection for yield in stress and non‐stress environments. Crop Sci. 21, 943–946.

[tpj14189-bib-0112] Saade, S. , Maurer, A. , Shahid, M. , Oakey, H. , Schmöckel, S.M. , Negrão, S. , Pillen, K. and Tester, M. (2016) Yield‐related salinity tolerance traits identified in a nested association mapping (NAM) population of wild barley. Sci. Rep. 6, 32586. 27585856 10.1038/srep32586 PMC5009332

[tpj14189-bib-0113] Sato, S. , Tabata, S. , Hirakawa, H. ***et** **al*** (2012) The tomato genome sequence provides insights into fleshy fruit evolution. Nature, 485, 635–641. 22660326 10.1038/nature11119 PMC3378239

[tpj14189-bib-0114] Scheben, A. , Wolter, F. , Batley, J. , Puchta, H. and Edwards, D. (2017) Towards CRISPR/Cas crops ‐ bringing together genomics and genome editing. New Phytol. 216, 682–698. 28762506 10.1111/nph.14702

[tpj14189-bib-0115] Schilling, R.K. , Marschner, P. , Shavrukov, Y. , Berger, B. , Tester, M. , Roy, S.J. and Plett, D.C. (2014) Expression of the Arabidopsis vacuolar H plus ‐ pyrophosphatase gene (AVP1) improves the shoot biomass of transgenic barley and increases grain yield in a saline field. Plant Biotechnol. J. 12, 378–386. 24261956 10.1111/pbi.12145

[tpj14189-bib-0116] Schnable, P.S. (2012) The B73 maize genome: complexity, diversity, and dynamics (November, pg 1112, 2009). Science, 337, 1040–1040. 10.1126/science.1178534 19965430

[tpj14189-bib-0117] Segura, V. , Vilhjalmsson, B.J. , Platt, A. , Korte, A. , Seren, U. , Long, Q. and Nordborg, M. (2012) An efficient multi‐locus mixed‐model approach for genome‐wide association studies in structured populations. Nat. Genet. 44, 825–U144. 22706313 10.1038/ng.2314 PMC3386481

[tpj14189-bib-0118] Servin, B. and Stephens, M. (2007) Imputation‐based analysis of association studies: candidate regions and quantitative traits. PLoS Genet. 3, e114. 17676998 10.1371/journal.pgen.0030114 PMC1934390

[tpj14189-bib-0119] Shikha, M. , Kanika, A. , Rao, A.R. , Mallikarjuna, M.G. , Gupta, H.S. and Nepolean, T. (2017) Genomic selection for drought tolerance using genome‐wide SNPs in maize. Front. Plant Sci. 8, 550. 28484471 10.3389/fpls.2017.00550 PMC5399777

[tpj14189-bib-0120] Silva, R.M.D. , Stafuzza, N.B. , Fragomeni, B.D. ***et** **al*** (2017) Genome‐wide association study for carcass traits in an experimental Nelore cattle population. PLoS ONE, 12, e0169860. 28118362 10.1371/journal.pone.0169860 PMC5261778

[tpj14189-bib-0121] Singh, A. , Ganapathysubramanian, B. , Singh, A.K. and Sarkar, S. (2016) Machine learning for high‐throughput stress phenotyping in plants. Trends Plant Sci. 21, 110–124. 26651918 10.1016/j.tplants.2015.10.015

[tpj14189-bib-0122] Sirault, X.R.R. , James, R.A. and Furbank, R.T. (2009) A new screening method for osmotic component of salinity tolerance in cereals using infrared thermography. Funct. Plant Biol. 36, 970–977. 10.1071/FP09182 32688708

[tpj14189-bib-0123] Spindel, J. , Begum, H. , Akdemir, D. , Virk, P. , Collard, B. , Redoña, E. , Atlin, G. , Jannink, J.‐L. and McCouch, S.R. (2015) Genomic selection and association mapping in rice (*Oryza sativa*): effect of trait genetic architecture, training population composition, marker number and statistical model on accuracy of rice genomic selection in elite, tropical rice breeding lines. PLoS Genet. 11, e1004982. 25689273 10.1371/journal.pgen.1004982 PMC4334555

[tpj14189-bib-0124] Struss, D. and Plieske, J. (1998) The use of microsatellite markers for detection of genetic diversity in barley populations. Theor. Appl. Genet. 97, 308–315.

[tpj14189-bib-0125] Sytar, O. , Brestic, M. , Zivcak, M. , Olsovska, K. , Kovar, M. , Shao, H.B. and He, X.L. (2017) Applying hyperspectral imaging to explore natural plant diversity towards improving salt stress tolerance. Sci. Total Environ. 578, 90–99. 27524726 10.1016/j.scitotenv.2016.08.014

[tpj14189-bib-0126] Tal, M. and Shannon, M.C. (1983) Salt tolerance in the wild relatives of the cultivated tomato: responses of Lycopersicon esculentum, L. cheesmanii, L. peruvianum, Solanum pennellii and F1 hybrids to high salinity. Aust. J. Plant Physiol. 10, 109–117.

[tpj14189-bib-0127] Tanger, P. , Klassen, S. , Mojica, J.P. ***et** **al*** (2017) Field‐based high throughput phenotyping rapidly identifies genomic regions controlling yield components in rice. Sci. Rep. 7, 42839. 28220807 10.1038/srep42839 PMC5318881

[tpj14189-bib-0128] Tanksley, S.D. and McCouch, S.R. (1997) Seed banks and molecular maps: unlocking genetic potential from the wild. Science, 277, 1063–1066. 9262467 10.1126/science.277.5329.1063

[tpj14189-bib-0129] Temnykh, S. , Declerck, G. , Lukashova, A. , Lipovich, L. , Cartinhour, S. and McCouch, S. (2001) Computational and experimental analysis of microsatellites in rice (Oryza sativa L.): frequency, length variation, transposon associations, and genetic marker potential. Genome Res. 11, 1441–1452. 11483586 10.1101/gr.184001 PMC311097

[tpj14189-bib-0130] Thomson, M.J. , Singh, N. , Dwiyanti, M.S. ***et** **al*** (2017) Large‐scale deployment of a rice 6 K SNP array for genetics and breeding applications. Rice, 10, 40. 28856618 10.1186/s12284-017-0181-2 PMC5577349

[tpj14189-bib-0131] Tipping, M.E. (2001) Sparse Bayesian learning and the relevance vector machine. J. Mach. Learn. Res. 3, 211–214.

[tpj14189-bib-0132] Tovar, J.C. , Hoyer, J.S. , Lin, A. ***et** **al*** (2018) Raspberry Pi‐powered imaging for plant phenotyping. Appl. Plant Sci. 6, e1031. 29732261 10.1002/aps3.1031 PMC5895192

[tpj14189-bib-0133] Tsaftaris, S.A. and Noutsos, C. (2009) Plant phenotyping with low cost digital cameras and image analytics In Information Technologies in Environmental Engineering, Environmental Science and Engineering. Heidelberg: Springer, pp. 238–251.

[tpj14189-bib-0134] Tschiersch, H. , Junker, A. , Meyer, R.C. and Altmann, T. (2017) Establishment of integrated protocols for automated high throughput kinetic chlorophyll fluorescence analyses. Plant Methods, 13, 54. 28690669 10.1186/s13007-017-0204-4 PMC5496596

[tpj14189-bib-0135] Tsujimoto, H. , Sohail, Q. and Matsuoka, Y. (2015) Broadening the Genetic Diversity of Common and Durum Wheat for Abiotic Stress Tolerance Breeding. Advances in Wheat Genetics: From Genome to Field. Tokyo: Springer Japan.

[tpj14189-bib-0136] Tu Anh, T. , Khanh, T. , Dat, T. and Xuan, T. (2018) Identification of phenotypic variation and genetic diversity in rice (Oryza sativa L.) mutants. Agriculture, 8, 30.

[tpj14189-bib-0137] Ubbens, J. , Cieslak, M. , Prusinkiewicz, P. and Stavness, I. (2018) The use of plant models in deep learning: an application to leaf counting in rosette plants. Plant Methods, 14, 6. 29375647 10.1186/s13007-018-0273-z PMC5773030

[tpj14189-bib-0138] UN Water and Energy . (2014) The United Nations World Water Development Report 2014. Paris: UNESCO.

[tpj14189-bib-0139] Verzilli, C. , Shah, T. , Casas, J.P. ***et** **al*** (2008) Bayesian meta‐analysis of genetic association studies with different sets of markers. Am. J. Hum. Genet. 82, 859–872. 18394581 10.1016/j.ajhg.2008.01.016 PMC2665011

[tpj14189-bib-0140] Vujkovic, M. , Aplenc, R. , Alonzo, T.A. , Gamis, A.S. and Li, Y. (2016) Comparing analytic methods for longitudinal GWAS and a case‐study evaluating chemotherapy course length in pediatric AML. A report from the children's oncology group. Front. Genet. 7, 139. 27547214 10.3389/fgene.2016.00139 PMC4974249

[tpj14189-bib-0141] Wakefield, J. (2007) A Bayesian measure of the probability of false discovery in genetic epidemiology studies. Am. J. Hum. Genet. 81, 208–227. 17668372 10.1086/519024 PMC1950810

[tpj14189-bib-0142] Waldmann, P. , Meszaros, G. , Gredler, B. , Fuerst, C. and Solkner, J. (2014) Evaluation of the lasso and the elastic net in genome‐wide association studies (vol 4, 2013). Front. Genet. 5, 270. 24363662 10.3389/fgene.2013.00270 PMC3850240

[tpj14189-bib-0143] Wan, H.P. , Chen, L.L. , Guo, J.B. , Li, Q. , Wen, J. , Yi, B. , Ma, C.Z. , Tu, J.X. , Fu, T.D. and Shen, J.X. (2017) Genome‐wide association study reveals the genetic architecture underlying salt tolerance‐related traits in rapeseed (Brassica napus L.). Front. Plant Sci. 8, 593. 28491067 10.3389/fpls.2017.00593 PMC5405135

[tpj14189-bib-0144] Wang, D. , Eskridge, K.M. and Crossa, J. (2011) Identifying QTLs and epistasis in structured plant populations using adaptive mixed LASSO. J. Agric. Biol. Environ. Stat. 16, 170–184.

[tpj14189-bib-0145] Wang, H. , Misztal, I. , Aguilar, I. , Legarra, A. and Muir, W.M. (2012) Genome‐wide association mapping including phenotypes from relatives without genotypes. Genet. Res. 94, 73–83. 10.1017/S0016672312000274 22624567

[tpj14189-bib-0146] Wang, C. , Hu, S. , Gardner, C. and Lübberstedt, T. (2017) Emerging avenues for utilization of exotic germplasm. Trends Plant Sci. 22, 624–637. 28476651 10.1016/j.tplants.2017.04.002

[tpj14189-bib-0147] Warburton, M.L. , Reif, J.C. , Frisch, M. ***et** **al*** (2008) Genetic diversity in CIMMYT nontemperate maize germplasm: landraces, open pollinated varieties, and inbred lines. Crop Sci. 48, 617.

[tpj14189-bib-0148] Watson, A. , Ghosh, S. , Williams, M.J. ***et** **al*** (2018) Speed breeding is a powerful tool to accelerate crop research and breeding. Nat. Plants, 4, 23–29. 29292376 10.1038/s41477-017-0083-8

[tpj14189-bib-0149] Whitt, S.R. , Wilson, L.M. , Tenaillon, M.I. , Gaut, B.S. and Buckler, E.S. (2002) Genetic diversity and selection in the maize starch pathway. Proc. Natl Acad. Sci. USA, 99, 12959–12962. 12244216 10.1073/pnas.202476999 PMC130568

[tpj14189-bib-0150] Wu, P. , Yang, Q. , Wang, K. ***et** **al*** (2018) Single step genome‐wide association studies based on genotyping by sequence data reveals novel loci for the litter traits of domestic pigs. Genomics, 110, 171–179. 28943389 10.1016/j.ygeno.2017.09.009

[tpj14189-bib-0151] Xu, X. , Liu, X. , Ge, S. ***et** **al*** (2012) Resequencing 50 accessions of cultivated and wild rice yields markers for identifying agronomically important genes. Nat. Biotechnol. 30, 105–U157. 10.1038/nbt.2050 22158310

[tpj14189-bib-0152] Yamaguchi, T. and Blumwald, E. (2005) Developing salt‐tolerant crop plants: challenges and opportunities. Trends Plant Sci. 10, 615–620. 16280254 10.1016/j.tplants.2005.10.002

[tpj14189-bib-0153] Yi, N.J. and Xu, S.H. (2008) Bayesian LASSO for quantitative trait loci mapping. Genetics, 179, 1045–1055. 18505874 10.1534/genetics.107.085589 PMC2429858

[tpj14189-bib-0154] Yin, K. , Gao, C. and Qiu, J.‐L. (2017) Progress and prospects in plant genome editing. Nat. Plants, 3, 17107. 28758991 10.1038/nplants.2017.107

[tpj14189-bib-0155] Zeng, A. , Chen, P. , Korth, K. , Hancock, F. , Pereira, A. , Brye, K. , Wu, C. and Shi, A. (2017) Genome‐wide association study (GWAS) of salt tolerance in worldwide soybean germplasm lines. Mol. Breeding, 37, 30.

[tpj14189-bib-0156] Zhang, Z. , Ersoz, E. , Lai, C.Q. ***et** **al*** (2010) Mixed linear model approach adapted for genome‐wide association studies. Nat. Genet. 42, 355–360. 20208535 10.1038/ng.546 PMC2931336

[tpj14189-bib-0157] Zhang, H. , Mittal, N. , Leamy, L.J. , Barazani, O. and Song, B.‐H. (2017) Back into the wild‐Apply untapped genetic diversity of wild relatives for crop improvement. Evol. Appl. 10, 5–24. 28035232 10.1111/eva.12434 PMC5192947

[tpj14189-bib-0158] Zhao, K.Y. , Aranzana, M.J. , Kim, S. ***et** **al*** (2007) An Arabidopsis example of association mapping in structured samples. PLoS Genet. 3, e4. 17238287 10.1371/journal.pgen.0030004 PMC1779303

[tpj14189-bib-0159] Zhao, Q. , Feng, Q. , Lu, H.Y. ***et** **al*** (2018) Pan‐genome analysis highlights the extent of genomic variation in cultivated and wild rice. Nat. Genet. 50, 279–284. 10.1038/s41588-018-0041-z 29335547

[tpj14189-bib-0160] Zheng, H. , Wang, H. , Yang, H. , Wu, J. , Shi, B. , Cai, R. , Xu, Y. , Wu, A. and Luo, L. (2013) Genetic diversity and molecular evolution of Chinese waxy maize germplasm. PLoS ONE, 8, e66606. 23818949 10.1371/journal.pone.0066606 PMC3688585

[tpj14189-bib-0161] Zhou, X. and Stephens, M. (2012) Genome‐wide efficient mixed‐model analysis for association studies. Nat. Genet. 44, 821–824. 22706312 10.1038/ng.2310 PMC3386377

[tpj14189-bib-0162] Zhou, X. , Carbonetto, P. and Stephens, M. (2013) Polygenic modeling with Bayesian sparse linear mixed models. PLoS Genet. 9, e1003264. 23408905 10.1371/journal.pgen.1003264 PMC3567190

[tpj14189-bib-0163] Zou, C.S. , Chen, A.J. , Xiao, L.H. ***et** **al*** (2017) A high‐quality genome assembly of quinoa provides insights into the molecular basis of salt bladder‐based salinity tolerance and the exceptional nutritional value. Cell Res. 27, 1327–1340. 28994416 10.1038/cr.2017.124 PMC5674158

